# The validity of self-rated health as a measure of health status among young military personnel: evidence from a cross-sectional survey

**DOI:** 10.1186/1477-7525-4-57

**Published:** 2006-08-29

**Authors:** Christopher K Haddock, Walker SC Poston, Sara A Pyle, Robert C Klesges, Mark W Vander Weg, Alan Peterson, Margaret Debon

**Affiliations:** 1 School of Medicine, University of Missouri-Kansas City, 2411 Holmes Street, Room MC-M03, Kansas City, MO 64110, USA; 2 Departments of Preventive Medicine and Family Medicine, Kansas City University of Medicine and Biosciences, 1750 Independence Avenue, Kansas City, MO 64106, USA; 3 Department of Preventive Medicine, St. Jude's Hospital, 66 N. Pauline, Suite 633, Memphis, TN 38163, USA; 4 Department of Internal Medicine, University of Iowa, 200 Hawkins Drive, Iowa City, IA 52241, USA; 5 Department of Psychiatry, University of Texas Health Sciences Center, 3939 Medical Drive, San Antonio, TX 78229, USA; 6 Health Sciences Center, University of Tennessee, 5050 Poplar Avenue, Suite 1800, Memphis, TN 38157, USA

## Abstract

**Background:**

Single item questions about self ratings of overall health status are widely used in both military and civilian surveys. Limited information is available to date that examines what relationships exist between self-rated health, health status and health related behaviors among relatively young, healthy individuals.

**Methods:**

The current study uses the population of active duty United States Air Force recruits (N = 31,108). Participants completed surveys that asked about health behaviors and health states and were rated their health on a continuum from poor to excellent.

**Results:**

Ratings of health were consistently lower for those who used tobacco (F = 241.7, p < .001), reported binge drinking (F = 69.0, p < .001), reported drinking and driving (F = 19.4, p < .001), reported taking health risks (F = 109.4, p < .001), were depressed (F = 256.1, p < .001) and were overweight (F = 39.5, p < .001).

**Conclusion:**

Given the consistent relationship between self-rated overall health and factors important to military health and fitness, self-rated health appears to be a valid measure of health status among young military troops.

## Background

Single item self-assessments of health are the most widely used measures of health status [1]. These self-assessments are used in many national surveys in the US, such as the National Health Interview Survey [2], National Health and Nutrition Examination Survey [3], and the Behavioral Risk Factor Surveillance System [4]. Self-rated health has been shown to be related to a number of important medical endpoints, such as health risk behaviors, disease states, disability, and mortality [1,5,6]. Self-ratings of health independently predict health parameters when compared to clinical evaluations and are sensitive to changes in health status [5,7].

The United States (US) Military and its healthcare plan, TRICARE, use a variety of health self-reporting surveys to assess the health status of military members and other beneficiaries. These questionnaires are used to provide data on the health and healthcare needs of all military healthcare beneficiaries and to target specific health issues. For instance, the Health Care Survey of DoD Beneficiaries [8] assesses a broad range of healthcare issues such as the use of preventive services while the Health Enrollment Assessment Review (HEAR) was developed to identify the health status of the military population [9]. In contrast, the Pre- and Post Deployment Health Assessment surveys [10] are used to monitor the health status of those military members deployed to overseas locations. One ubiquitous measure on these surveys is a single-item which asks respondents to rate their overall health. An implicit assumption of this item is that an individual's self-assessment of overall health provides a valid representation of the individual's health status [1].

Despite the widespread use of self-assessments of overall health among military personnel, research is lacking regarding the ability of these items to predict health status among this relatively young, healthy population. In the one study to date, Trump and colleagues [11] found that self-reports of low health status were related to higher health needs after military deployment. Additional data are needed so that military leaders can appropriately use data regarding a military member's assessment of their own health. In this study, we used key health behaviors (e.g., tobacco, alcohol and drunk driving habits) to examine the validity of self-rated overall health as a measure of health status in an entire population (N = 31,108) of active duty recruits entering the US Air Force. In addition, prospective data were used to determine whether self-ratings of health were predictive of two important longitudinal outcomes among recruits, smoking initiation and discharge from military service. It is expected that, even among relatively healthy and young individuals, the relationship between self ratings of overall health will be consistent with previous findings with other populations [5,7]. Validating a brief measure of overall health status for young troops may result in a useful population health measure for the military and similar organizations.

## Methods

### Overview of parent project

This study was conducted as part of a study of a large randomized tobacco control trial among U.S. Air Force recruits. In this investigation, those recruits entering the United States Air Force (USAF) Basic Military Training (BMT) who were to be active duty and entered the enlisted ranks of the USAF from October 1999 to September 2000 completed a comprehensive health questionnaire (N = 31,108).

### Participants

Table 1 presents demographic characteristics of the population of recruits. Average age of the participants was 19.95 years (SD = 1.99) and 25.2% were female. Most of the recruits were not married (> 90%) and approximately one-fifth (21.1%) had attended at least some college. Minority representation was high among all participants, particularly among females where almost 26% were African-American (n = 2,027) and 10.9% were Hispanic (n = 850). Current smokers (i.e., those who had smoked up to the start of BMT) comprised 37.7% of the participants.

**Table 1:** Demographics

**Sample (N)**	**"Would you say your overall physical health is..." (%)**					**Mean**^**†**^	**F(p^**‡**^)**
			
	**Poor**	**Fair**	**Good**	**Very Good**	**Excellent**		
**All Recruits **(31,108)	0.8	11.6	43.0	35.0	9.6	3.40	
**Gender**							141.8
Males (23,282)	0.7	10.4	41.1	37.2	10.6	3.45	(<.001)
Females (7,826)	1.0	15.3	48.7	28.3	6.7	3.23	
**Ethnicity/Race**							8.5
Asian/Pacific Islander (1,255)	1.0	14.3	44.9	30.0	9.8	3.33	(<.001)
African-American (5,826)	0.9	11.2	38.5	36.3	13.2	3.48	
Hispanic (3,129)	0.6	11.4	38.8	36.7	12.4	3.49	
White (19,751)	0.7	11.6	45.0	34.6	8.0	3.36	
Native American (234)	1.3	10.7	42.3	35.0	10.7	3.43	
Other (912)	0.5	11.0	41.4	36.3	10.7	3.46	
**Marital Status**							5.6
Married (2,931)	1.0	12.7	46.3	31.3	8.6	3.34	(.018)
Not Married (28,177)	0.7	11.5	42.7	35.4	9.7	3.40	

Note: percentages may not add to 100 due to rounding. ^†^Mean rating based on assigning values of 1 = Poor through 5 = Excellent health. ^‡^p-value

### Baseline assessment methods

In the second week of BMT, trainees completed the baseline assessment questionnaire. Administration was conducted in a group setting in "flights" (the Air Force equivalent of platoons) of approximately 50 individuals per flight in a classroom setting. Participants received verbal instruction on how to complete the health questionnaire and the research staff checked each questionnaire for completeness before each flight was dismissed. The study was approved by the review boards of the participating Universities (University of Memphis, University of Minnesota, and University of Missouri – Kansas City) and by the Wilford Hall Medical Center's Clinical Investigations Directorate. Participants completed a written consent document prior to the completion of the baseline questionnaire.

### Twelve-month follow-up assessment methods

Twelve-month follow-up forms were sent through the mail to all active duty participants who reported being current or former tobacco users at baseline. A random sample of 17 never and experimental smokers from each flight also were initially selected for follow-up. Due to a greater than anticipated number of discharges from the Air Force during the one-year follow-up, the percentage of nonsmokers that were sampled was subsequently increased by 13% in order to ensure adequate statistical power in the parent study. Those who did not respond to either of two mailings were contacted by telephone to complete the follow-up survey. The average follow-up rate among those that were randomly selected for follow-up was 89.9%. This follow-up rate is slightly lower than our previous study as our follow-up interval covered the period of the 9/11 terrorist attack and the Air Force for several months solely focused on mobilizing for war in Afghanistan and Iraq as well as homeland security. During this time, a large percentage of our participants were literally moved overnight and many were moved to "undisclosed locations" (meaning their location was now classified), making tracking these participants impossible.

### Definition of key study variables

A 67-item baseline questionnaire was developed for use in the parent project. Items were selected from previous surveys, including those used in prior studies with USAF recruits [12,13]. Self-reported health status was assessed using the single-item question "would you say your overall physical health is:" followed by five possible responses; "poor", "fair", "good", "very good", and "excellent". The validity of this item as a measure of health status was assessed using health behaviors which are traditionally important indicators of military fitness for duty such as smoking, alcohol use, depressed mood, taking risks with one's health, and weight status [14]. In addition, the relationship between self-rated health and discharge from the military was examined. The following is a description of key variables used to validate self-rated health ratings:

### Smoking status

Smoking status was assessed with the following item: What was your history of cigarette smoking (not including clove cigarettes) just prior to Basic Military Training? Possible responses were: (1) I have never smoked, not even a puff; (2) I have only smoked on one or two occasions in the past; (3) I smoked regularly (at least once per day), but quit in the past 6 months; (4) I smoked regularly (at least once per day), but quit between 6 months and one year ago; (5) I smoked regularly (at least once per day), but quit more than a year ago; (6) I smoked, but not every day; and (7) I smoked every day. Participants selecting responses 1 or 2 were termed "Never Smokers" (i.e., never smoking regularly), participants selecting responses 3, 4, or 5 were "Ex-Smokers, while responses 6 and 7 defined "Current Smokers".

### Intentions to smoke after BMT

Given that all troops were smoke-free during BMT, participants were asked "Once you get out of Basic Military Training, which of these best describes you:" with the following possible responses: "plan to stay quit", "thinking about staying quit", "do not plan to stay quit".

### Alcohol abuse

Binge drinking was assessed with the following item: "Including all types of alcoholic beverages, how many times during the 30 days prior to BMT did you have 5 or more drinks on one occasion?" Those who reported one or more binge drinking episodes were categorized as "Yes": all other participant responses were labeled "No". Drinking and driving was assessed with the item: "In the 30 days prior to BMT, how many times have you driven a motor vehicle after drinking an alcoholic beverage?" and was scored identically to the binge drinking item.

### Weight status

Weight status was assessed using BMI. BMI is defined as the ratio of weight measured in kilograms divided by the square of height measured in meters. BMI is a simple, easy to use, and cost-effective screening method because it is highly correlated with various measures of body fat [15]. Overweight is typically diagnosed at a BMI greater than 25 and obesity at 30. Underweight is defined as a BMI of below 18. For this study, underweight was defined by a BMI of less than 18, normal weight by a BMI between 18.0 and 24.9, and overweight/obese by a BMI greater than or equal to 25.0. Overweight and obesity were aggregated into one category because of the low prevalence of BMI's above 30 (0.4%) in the USAF sample due to weight and fitness requirements for entry into military service.

### Health risk taking

Proclivity to take health risks was assessed with the item "I like to take health risks (e.g., abusing my body, being inactive, overeating, driving fast)." Participants responded to this item on a 5-point Likert Scale from "Strongly Agree" to "Strongly Disagree". For analytical purposes, responses were divided between those who were inclined to take health risks (i.e., "Strongly Agree" or "Agree") and those disinclined to take health risks (i.e., "Neutral", "Disagree", or "Agree").

### Depressed mood

Depressed mood was measured with the following item: "I feel sad and blue most of the time." Participants responded on a 5-point Likert scale from "Strongly Agree" to "Strongly Disagree." Responses were divided between those who reported low mood (i.e., "Strongly Agree" or "Agree") and those not reporting low mood (i.e., "Neutral", "Disagree", or "Agree").

### Longitudinal outcomes

Two key longitudinal factors were used to validate self-ratings of health: smoking status and discharge from the military. Smoking status at the one-year follow up was assessed using a 7-day point prevalence analysis [16]. Discharge was assessed both after BMT and after technical training school, the second level training after BMT, and before a participant's permanent duty assignment.

### Approach to statistical analyses

In order to explore differences in self-rated health based on demographic characteristics, participants were stratified based on gender, ethnicity and marital status. Groupings were made in relation to current and predicted health behaviors (e.g. drinking, smoking) to determine if self-rated health was related to perceived and prospective actions. Participants were then stratified based on their reported smoking status at entry to BMT (current, former, never) and comparisons were made between those who were and were not smoking at the one year follow-up in order to determine the relationship between smoking initiation or relapse and perceived health. Finally, comparisons were made between those who were not discharged, those who were discharged during BMT and those who were discharged during technical training to examine whether perceived health relates to early discharge from the military. Using SPSS 13.0 data were presented in two complementary forms. First, group means were compared using a one-way ANOVA. Second, given the unique emphasis on health and readiness in the military, comparisons for health behaviors, weight, and discharge were made on the proportion of participants who placed themselves at the top of the self-rated health – "Very Good" or "Excellent" (henceforth referred to as "VG/E" health) using logistic regression analysis. This approach is consistent with several previously published studies which examine predictors of extreme ratings on self-rated health questions [11].

## Results

### Demographics characteristics

Table 1 contains demographic information about the sample as well as comparisons between groups. Overall, men rated perceiving their physical health as significantly better then women (F = 141.8, p < .001). Nearly half of men (48%) compared to about one-third of women (35%) rated their physical health as "very good" or "excellent". Significant differences also exist in overall mean ratings between ethnic groups (F = 8.5, p < .001). African-Americans (M = 3.48) and Hispanics (M = 3.49) reported the highest average perceived health self-ratings while Asian/Pacific Islanders (M = 3.33) reported perceiving their physical health as worst. Those who were not married reported significantly better health than those who were (F = 5.6; p = 0.018).

### Cigarette smoking

Table 2 presents comparisons of the sample based on reported health behaviors and predictions about future health behaviors. Not surprisingly, those who had never smoked reported the best physical health while those who were current smokers at the beginning of BMT reported the worst health (F = 241.7, p < .001). Differences among smoking status categories were particularly noticeable when looking at the percent of participants who reported VG/E health ratings. Smokers were 31% less likely (p < .001; table 4) and ex smokers were 58% less likely (p < .001) to rate their health as VG/E compared to never smokers. Mean physical health ratings for those who predicted that they would smoke or who were not sure whether they would smoke after BMT were low compared with those who were sure they would not smoke after BMT (F = 190.3, p < .001). As with smoking status, differences among the three smoking intention groups were particularly large when looking at participants who rated their health as VG/E. Compared to participants who predicted they would not smoke after BMT, those who were unsure whether they would smoke were 23% less likely (p < .001) while those reporting they would smoke were 18% less likely (p = .001) to rate their health as VG/E.

**Table 2:** Self Ratings of Overall Health and Indicators of Health Status

**Sample (N)**	**"Would you say your overall physical health is..." (%)**					**Mean**^**†**^	**F(p^**‡**^)**
			
	**Poor**	**Fair**	**Good**	**Very Good**	**Excellent**		
**Smoking Status**							241.7
Current Smoker (10,163)	1.1	16.3	50.8	27.4	4.3	3.15	(<.001)
Ex Smoker (2,394)	0.8	13.3	44.7	34.0	7.1	3.33	
Never Smoker (18,549)	0.5	8.8	38.5	39.3	12.8	3.54	
**I Will Smoke After BMT**							190.3
Yes (5,977)	1.3	16.0	49.0	28.5	5.1	3.18	(<.001)
Not Sure (5,694)	0.7	15.1	49.9	29.5	4.8	3.21	
No (19,437)	0.6	9.2	39.2	38.6	12.4	3.52	
**Binge Drinking (Past 30 Days)**							69.0
Yes (11,991)	0.8	13.5	45.4	33.3	7.0	3.31	(<.001)
No (19,117)	0.7	10.4	41.5	36.1	11.3	3.45	
**Driving After Drinking (Past 30 Days)**							19.4
Yes (2,095)	1.7	13.9	46.1	32.8	5.4	3.26	(<.001)
No (29,013)	0.7	11.4	42.8	35.1	9.9	3.41	
**Like to Take Health Risks**							109.4
Yes (1,131)	5.2	22.7	40.1	25.5	6.4	2.96	(<.001)
No (29,977)	0.6	11.2	43.1	35.4	9.7	3.41	
**Feel Sad or Blue Most of the Time**							256.1
Yes (1,680)	5.2	27.9	42.1	19.9	4.9	2.85	(<.001)
No (29,428)	0.5	10.7	43.1	35.9	9.9	3.43	
**Weight Status (BMI)**							39.5
Underweight (1,246)	0.9	14.6	43.3	32.7	8.5	3.33	(<.001)
Normal Weight (22,955)	0.7	10.5	41.7	36.7	10.4	3.44	
Overweight/Obese (6,878)	1.0	14.9	47.3	29.6	7.1	3.27	

Note: percentages may not add to 100 due to rounding. ^†^Mean rating based on assigning values of 1 = Poor through 5 = Excellent health. ^‡^p-value

**Table 3:** Self ratings of overall physical health, smoking at one-year, and discharge

**Sample (N)**	**"Would you say your overall physical health is..." (%)**					**Mean**^**†**^	**F(p^**‡**^)**
			
	**Poor**	**Fair**	**Good**	**Very Good**	**Excellent**		
**Current Smokers**							8.53
Not Smoking (2,054)	0.9	14.9	48.4	30.5	5.3	3.24	(.004)
Smoking (5,734)	1.0	16.4	52.5	26.2	3.9	3.14	
**Ex Smokers**							7.76
Not Smoking (678)	0.1	11.1	42.8	37.5	8.6	3.43	(.005)
Smoking (444)	1.8	12.6	4.71	31.5	7.0	3.29	
**Never Smokers**							1.76
Not Smoking (10,516)	0.4	8.3	38.5	39.7	12.9	3.55	(.185)
Smoking (1,246)	0.5	9.5	40.5	38.2	11.3	3.50	
**Discharge**							19.53
Not Discharged (29,821)	0.7	11.3	43.0	35.3	9.7	3.41	(<.001)
Discharged During BMT (939)	2.8	20.1	45.3	25.2	6.6	3.13	
Discharged During Tech Training (348)	2.0	17.0	42.5	32.2	6.3	3.24	

Note: percentages may not add to 100 due to rounding. ^†^Mean rating based on assigning values of 1 = Poor through 5 = Excellent health. ^‡^p-value

**Table 4:** Logistic regressions predicting participants reporting health as Very Good/Good/Excellent (VG/E)

**Variable**	**Odds Ratio**	**p value**
**Smoking Status**		
Non Smokers	1.00	
Ex Smokers	0.42	<.001
Current Smokers	0.69	<.001
**Predicted Smoking Status After BMT**		
Do not believe they will smoke	1.00	
Unsure if they will smoke	0.77	<.001
Will smoke	0.82	<.001
**Binge Drinking**		
No binge drinking episodes in last 30 days	1.00	
One or more binge drinking episodes in last 30 days	0.75	<.001
**Drunk Driving**		
No driving while drinking in past 30 days	1.00	
One or more drinking and driving episodes in past 30 days	0.76	<.001
**Weight Status**		
Normal weight	1.00	
Underweight	0.79	<.001
Overweight	0.65	<.001
**Depressed Mood**		
No episodes of depressed mood	1.00	
Episodes of depressed mood	0.39	<.001
**Health Risks**		
Reported not taking health risks	1.00	
Reported taking health risks	0.82	<.001

### Alcohol abuse

Those participants who reported they had not had a drinking binge within the last 30 days reported significantly better physical health than binge drinkers (F = 69.0, p < .001). Similarly, binge drinkers were 25% less likely (p < .001) to rate their physical health as VG/E compared to those who did not binge drink. Participants who had not driven after drinking reported better physical health than those who had (F = 19.4, p < .001). Also, those who reported driving while drinking were 25% less likely (p < .001) to report VG/E physical health compared to those who had not driven after drinking.

### Weight status

Weight status was significantly related to self-rated physical health (F = 39.5, p < .001) with those of normal weight reporting the best health, those classified as underweight second and those who were overweight reporting the worst self-rated health. When examining the proportion of VG/E physical health ratings by weight status, underweight participants were 21% less likely (p < .001) and overweight participants were 35% less likely (p < .001) to report VG/E health.

### Depressed mood and health risk taking

Both depressed mood (F = 256.1, p < .001) and health risk taking (F = 109.4, p < .001) demonstrated strong associations with self-rated physical health. Participants reporting depressed mood were 61% less likely (p < .001) to report VG/E physical health compared to those not reporting depressed mood. Similarly, those who reported liking to take health risks were 18% less likely (p < .001) to rate their physical health as VG/E compared to other participants.

### Smoking initiation/relapse and discharge

Of those who were smoking when they entered BMT, 74% had returned to smoking within a year and reported viewing their physical health as significantly worse than those who did not return to smoking (F = 8.53, p = .004; See Table 3). Those who returned to smoking were 23% less likely to report VG/E physical health then those who did not relapse (p < .001; see Table 5). Similarly, the 40% of former smokers at baseline who re-initiated smoking within a year of enlisting in the Air Force reported significantly worse physical health than their smoke-free peers (F = 7.76, p = .005). Those former smokers who reported re-initiating after BMT were 26% less likely than their peers to report VG/E physical health (p = .013). Among those who were not smoking at baseline, self ratings of physical health were not significantly different overall for those who had initiated smoking and those who had not at follow-up (F = 1.76; p = .185). However, those who had initiated smoking were slightly less likely to report VG/E physical health (OR = .88, p = .035). Significant differences also existed between participants who were discharged from the military and those who were not (F = 19.53; p < .001). Compared to those not discharged, participants discharged during BMT were 43% less likely (< .001) to report VG/E physical health and those discharged during technical training school were 24% less likely (p < .001) to report VG/E physical health.

**Table 5:** Logistic regressions predicting participants reporting health as Very Good or Excellent (VG/E), longitudinal data

**Variable**	**Odds Ratio**	**p value**
**Smoking Status of Smokers at Follow-up**		
Smokers who did not return to smoking	1.00	
Smokers who returned to smoking	0.77	<.001
**Smoking Status of Former Smokers at Follow-up**		
Former smokers at baseline who did not re-initiate smoking	1.00	
Former smokers at baseline who did re-initiate smoking	0.74	.013
**Smoking Status of Non-Smokers at Follow-up**		
Non smokers at baseline who did not begin smoking	1.00	
Non smokers at baseline who began smoking	0.88	.035
**Discharge**		
Not discharged	1.00	
Discharged during BMT	0.57	<.001
Discharged during technical training	0.76	<.001

## Discussion

This study examined the validity of self-rated overall physical health as it relates to health status in a population of young military recruits. Using a single item ("would you say your overall physical health is:"), troops rated their health on a 5-point scale from poor to excellent. A consistent pattern emerged where troops who reported negative health behaviors (e.g. smoking, drinking and driving, excessive alcohol use) also reported poorer overall self-rated health. Similarly, those who predicted they would initiate or experiment with negative health behaviors (e.g. smoking) or who reported a proclivity to take risks with their health reported poorer overall health. Overweight individuals described their health most negatively when compared with normal weight and underweight individuals. Finally, troops who were discharged during either basic training or technical training school reported worse overall health. These results suggest that overall self-rated physical health is consistently associated with poorer health behaviors, a liking to take health risks, not maintaining a healthy weight, and being discharged from the military. Interestingly, it should be noted that the results in regard to SRH when stratified by ethnicity were different than found in previous literature [17]. While the current study found White and Asian/Pacific Islander participants reported the poorest health and Hispanic and African Americans the highest, the opposite has been true in previous studies. It is possible that this is related to the relative youth of the participants and that age distribution plays a significant role in SRH as it relates to ethnicity.

Strong relationships were also found between the proportion of participants who rated their physical health as "Very Good" or "Excellent" (VG/E) and health behaviors, weight status, and discharge. Consistently, participants with more problematic health behaviors (e.g., smoking, binge drinking), higher weight status, or who were discharged from the military were less likely to rate their overall physical health as VG/E. Results were particularly strong for smoking status, where smokers were almost two and one-half times less likely to rate their physical health as VG/E compared to never smokers. This is not surprising given the negative impact smoking has on health, even among young military troops [12,13,18]. The military has traditionally had a higher prevalence of smoking when compared to the civilian sector, which is likely to negatively impact both actual and perceived health of its troops. The relationship between VG/E ratings and depressed mood was also strong, with troops reporting depressed mood being 2.6 times less likely to rate their physical health as VG/E compared to non-depressed troops. Depression has been found to be a primary reason for mental health-related discharge for young military recruits and an important indicator of fitness for duty [19,20]. While a single item about depressed mood is not equivalent to a comprehensive review of depressive symptoms, the strong relationship found provides and interesting basis for future research.

Bailis and colleagues [7] speculate that overall self ratings of health can be explained through two different perspectives. Ratings may be the result of *spontaneous assessments *of health or may be the result of *enduring self-concepts*. The *spontaneous assessment *perspective posits that ratings of overall self rated health are developed based on health status at any given point in time and fluctuates as health status changes. Alternatively, the *enduring self-concept *perspective suggests that self ratings of health are based on a person's behavioral intentions, personal health practices, and a person's self concept. Results from the current study suggest that both perspectives of the development of ratings may be viable explanations because significant relationships were found between overall self-ratings of health and both reported health status and behavioral intentions for the future. However, the cross-sectional nature of the current study limits the conclusions that can be drawn at this time.

Given the consistent and sometimes strong relationship between self-rated overall physical health and factors important to military health and fitness, self-rated health appears to be a valid measure of health status among young military troops. The fact that this population is young, generally healthy, and has been screened for many medical and psychiatric conditions suggests that even in this unique group self-rated overall health provides potentially valuable health status data. The findings of this study are consistent with the one other study of self-rated health among military members which found that troops who rated their health as poor or fair were at significant risk for high use of health services after deployment compared to other troops [11]. The results are also consistent with a large civilian literature which demonstrates a relationship between self-rated general health and important medical endpoints [1,5,6]. Given its brevity and apparent validity as a marker for health and health behaviors, self-rated health may prove to be a useful tool for assessing health status among young military members.

Self-rated health data could provide at least two important benefits for military leaders. First, using self-rated health as a population screener will enable the military to better target preventive health interventions. It is difficult and costly to direct prevention efforts at all troops – so simple screening tools are needed to target resources. This study suggests that even a single-item assessment of health would provide useful information for military health planners. Second, self-rated health measures could help the military to profile the health of troops. If measures of self-rated health significantly change over time, reasons for the changes in population health could be identified. For instance, self-rated health measures could be used both pre- and post-deployment to help determine which individuals have significant changes in health status.

Although this study has many strengths, including assessment of an entire population of military recruits, there are limitations to the data presented. Assessing all recruits on a broad spectrum of health parameters required self-reports of all health outcomes. For most of the health issues presented in this study, self-reports are considered valid for population-level research. For instance, self-reports of both tobacco use [2,22,23] and weight status [24,25] have been found to be highly related to more objective assessments of each condition and are commonly used in national surveys such as the BRFSS [26,27]. However, it is still possible that social desirability may have influenced the findings. In addition, for the use of this study, self ratings of overall physical health were used to operationalize overall self-rated health. It should be noted that the question used asked about physical health rather than overall health that could have included other domains (e.g. mental health). Furthermore, while items selected have been used in previous research, not all items had available psychometric information. This study was only conducted in one military service. Whether these results generalize to other military branches, foreign military services, or related organizations (e.g., law enforcement recruits, fire fighters) is unknown. Also, it should be noted that the large sample size of this study may result in small effects reaching statistical significance.

In summary, a single-item self-assessment of health was consistently related to a variety of health parameters important to the military. Used at a population level, this brief health status measure may prove to be a useful tool for targeting health services to this unique population. Additional studies are needed, however, to determine if the results found in this study generalize to the other military branches or other security services. Additional research on the longitudinal relationship between overall self rated health and health risk factors may also prove useful. Research should also focus on the impact interventions focused on health behaviors and behavioral intentions have on overall self rated health. It is possible that overall self rated health status may serve as a viable measure of the efficacy of health interventions.

## Abbreviations

United States (US)

Health Enrollment Assessment Review (HEAR)

United States Air Force (USAF)

Basic Military Training (BMT)

"Very Good" or "Excellent" (VG/E)

## Competing interests

The author(s) declare that they have no competing interests.

## Authors' contributions

CKH was involved in project design and development. He was primarily responsible for the manuscript preparation, wrote a substantial portion of the manuscript and provided final approval of manuscript content. WSCP was involved in project design and development. He was involved in concept development and statistical design of the manuscript, was involved in background research and provided final approval of manuscript content. SAP was involved in manuscript development, assisted in background research, performed statistical analyses, participated in writing both the background and conclusions, and provided final approval of manuscript content. RCK was the principal investigator of the parent project. He oversaw instrument development, surveying and project completion. He assisted in developing the concept for this manuscript, provided expertise and final approval of manuscript content. MWV was instrumental in the parent project from which the data was collected. He assisted in instrument development, surveying and project completion. He assisted in developing the concept for this manuscript, provided expertise and final approval of manuscript content. AP was the primary military contact for this project. He assisted in project development and design as well as instrument development and design. He oversaw implementation of the project. He assisted in developing the concept for this manuscript, provided expertise and final approval of manuscript content. MD was instrumental in the parent project from which the data was collected. He assisted in instrument development, surveying and project completion. He assisted in developing the concept for this manuscript, provided expertise and final approval of manuscript content.
